# Structural insights into RNA polymerase III-mediated transcription termination through trapping poly-deoxythymidine

**DOI:** 10.1038/s41467-021-26402-9

**Published:** 2021-10-21

**Authors:** Haifeng Hou, Yan Li, Mo Wang, Aijun Liu, Zishuo Yu, Ke Chen, Dan Zhao, Yanhui Xu

**Affiliations:** 1 Fudan University Shanghai Cancer Center, Institutes of Biomedical Sciences, State Key Laboratory of Genetic Engineering, Shanghai Key Laboratory of Radiation Oncology, and Shanghai Key Laboratory of Medical Epigenetics, Shanghai Medical College of Fudan University, Shanghai, 200032 China; 2 The International Co-laboratory of Medical Epigenetics and Metabolism, Ministry of Science and Technology, China, Department of Systems Biology for Medicine, School of Basic Medical Sciences, Shanghai Medical College of Fudan University, Shanghai, 200032 China; 3 Human Phenome Institute, Collaborative Innovation Center of Genetics and Development, School of Life Sciences, Fudan University, Shanghai, 200433 China; 4 State Key Laboratory of Reproductive Regulation and Breeding of Grassland Livestock School of Life Sciences, Inner Mongolia University, Hohhot, 010070 P. R. China

**Keywords:** Structural biology, Transcriptional regulatory elements, Cryoelectron microscopy

## Abstract

Termination of the RNA polymerase III (Pol III)-mediated transcription requires the conversion of an elongation complex (EC) to a pre-termination complex (PTC) on poly-deoxythymidine (dT)-containing non-template strand, a mechanism distinct from Pol I and Pol II. Here, our in vitro transcription elongation assay showed that 5-7 dT-containing DNA template led to transcription termination of Pol III, but not Pol I or Pol II. We assembled human Pol III PTC on a 7 dT-containing DNA template and determined the structure at 3.6 Å resolution. The structure reveals that poly-dT are trapped in a narrow exit tunnel formed by RPC2. A hydrophobic gate of the exit tunnel separates the bases of two connected deoxythymidines and may prevent translocation of the non-template strand. The fork loop 2 stabilizes both template and non-template strands around the transcription fork, and may further prevent strand translocation. Our study shows that the Pol III-specific exit tunnel and FL2 allow for efficient translocation of non-poly-dT sequence during transcription elongation but trap poly-dT to promote DNA retention of Pol III, revealing molecular mechanism of poly-dT-dependent transcription termination of Pol III.

## Abstract (web summary)

Termination of eukaryotic RNA polymerase III (Pol III)-mediated transcription occurs when the polymerase reaches a stretch of four or more deoxythymidine nucleotides (poly-dT) on the non-template strand. Here, the authors present the 3.6 Å cryo-EM structure of a human Pol III pre-termination complex (PTC) that was assembled on a 7 dT-containing DNA template and discuss the mechanism of poly-dT-dependent transcription termination of Pol III.

## Introduction

The eukaryotic transcription mediated by RNA polymerases requires tight control throughout transcription initiation, elongation, and termination. As an obligatory step of transcription, termination is critical for proper maturation and release of transcripts, recycle of polymerases, and clearance of template DNA for subsequent transcription^1^. Dysregulation of transcription termination leads to a severe defect in gene expression and causes genetic diseases^2^.

Distinct from the relatively conserved catalytic mechanism, the process of transcription termination seems to be more diversified among RNA polymerases across species^3–6^. Despite the well characterized mechanism of prokaryotic transcription termination, it remains incompletely understood how transcription is properly terminated in eukaryotes^7^. Among the three mammalian RNA polymerases (Pol I, Pol II and Pol III), Pol I and Pol II require multipartite *cis*-regulatory elements and *trans*-acting factors to terminate the transcription^7^. By contrast, termination of Pol III-mediated transcription occurs when the polymerase reaches a stretch of more than three deoxythymidine nucleotides (poly-dT) on the non-template strand (strand^NT^), suggesting a Pol III-specific termination mechanism^8–13^.

In Pol III-mediated transcript, the productive elongation complex (EC) is converted into a transcriptionally active but metastable pre-termination complex (PTC)^12^, a key process of transcription termination. It has been reported that poly-dT in non-template strand initiates the termination of transcription-independent of other *cis*-regulatory elements or *trans*-acting factors. The average lengths of the poly-dT in canonical Pol III termination sites vary across species, with an average of 5–7 dTs in *Schizosaccharomyces pombe* (*S. pombe*), 6–9 dT in *Saccharomyces cerevisiae* (*S. cerevisiae*), and 4–5 dT in vertebrates. This unique termination mechanism is beneficial to the functions of Pol III, which transcribes a large amount of short essential transcripts^14,15^ and requires high efficiency during the transition from termination to reinitiation^16,17^.

Human Pol III consists of 17 subunits that are organized into a catalytic core (RPC1-2, RPAC1–2, RPABC1–5, and RPC10), a stalk module (RPC8-9), peripheral trimer (RPC3, RPC6, and RPC7) and a dimer module (RPC4-5)^18–23^. Previous studies reported the structures of yeast and human Pol III and the mechanisms of transcription initiation and elongation^20–29^. The structures revealed that the catalytic core of Pol III is similar to that of Pol I and Pol II, consistent with its conserved function in transcription elongation^19^. The three RNA polymerases also contain distantly related stalk subunits, A14/43, Rpb4/7, and C17/25 in yeast Pol I, Pol II, and Pol III, respectively. In addition, Pol I contains TFIIF-like heterodimer A49/34.5 and Pol III contains TFIIF-like heterodimer C37/53 and TFIIE-like heterotrimer C82/34/31^18^. These peripheral subunits play a regulatory role in transcription initiation and termination by directly interacting nucleic acids and/or involving in protein–protein interaction network^19^. The termination-reinitiation subcomplex (RPC4-RPC5-RPC10) of Pol III plays a critical role in regulating transcription termination^12,30–32^. Previous studies also showed that termination-defect mutations were mapped to residues 300–325 and 455–521 of the yeast RPC2 subunit. Despite these studies, it remains largely unknown how such a stretch of poly-dT on strand^NT^ could lead to effective transcription termination on Pol III but not on Pol I or Pol II.

Following our recent structural study of the human Pol III EC complex^21^, we reconstituted a mimetic PTC complex and determined the cryo-electron microscopy (EM) structure at 3.6 Å resolution. The structure indicates that poly-dT in strand^NT^ is trapped in a non-template strand exit tunnel (termed exit tunnel below). Translocation of this poly-dT is unfavorable due to the conformational restrain by the exit tunnel and the stabilization of the transcription fork. Our study provides structural insights into the transition from transcription elongation to pre-termination and reveals the molecular mechanism of poly-dT-dependent transcription termination of Pol III.

## Results

### Poly-dT guides transcription termination of Pol III

The recombinant human Pol III complex was transiently expressed in human embryonic kidney Expi293F cells and purified to homogeneity as previously reported^21^ (Supplementary Fig. 1a). To investigate the termination efficiency by poly-dT in vitro, we assembled RNA Pol II (pig endogenous Pol II)^33,34^ and Pol III elongation complexes on various RNA-DNA scaffolds (Supplementary Table 1), which we termed termination sequence 1 (TS1). These TS1 sequences contain the different lengths of the dT stretch, which were designated TS1^T1^, TS1^T3^, TS1^T5^, and TS1^T7^. Transcription elongation reactions were performed by the addition of nucleoside triphosphate (NTP) substrates to a final concentration of 1.25 mM each. As expected, Pol II could read-through all the TS1 templates without early termination (Supplementary Fig. 1b, lanes 2, 4, 6, and 8). By contrast, Pol III could read-through TS1^T1^ and TS1^T3^ (Supplementary Fig. 1b, lanes 3 and 5) but showed largely reduced read-through and obvious termination on TS1^T5^ and TS1^T7^ (Supplementary Fig. 1b, lanes 7 and 9), RNA products around 43 nt in length indicated with red box). These results indicate that poly-dT-containing DNA template (more than three continuous deoxythymidine) leads to Pol III (but not Pol II)-mediated transcription termination and the length of poly-dT is positively correlated to the termination efficiency. Notably, read-through by Pol III was also observed on TS1^T5^ and TS1^T7^ templates (Supplementary Fig. 1b, lanes 7 and 9), indicating dynamic equilibration between transcription elongation and termination.

### Structure determination of the human Pol III pre-termination complex

**Fig. 1:**
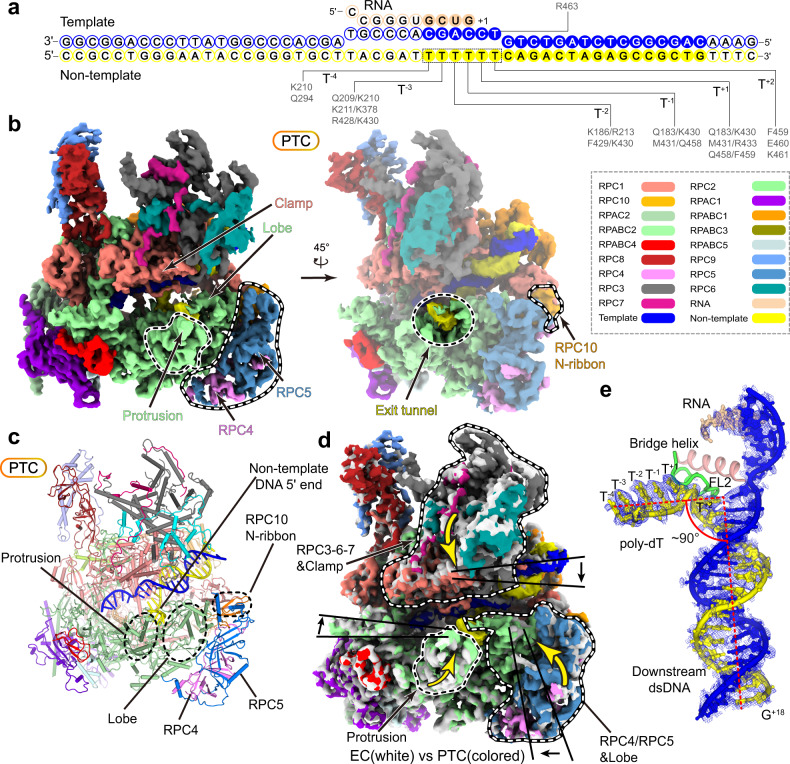
Cryo-EM structure of the human Pol III PTC. **a** Schematic diagram of the nucleic acid scaffold and its interactions with Pol III. RNA, template DNA, and non-template DNA are colored in wheat, yellow, and blue, respectively. The non-template strand is numbered relative to the ribonucleotide addition site (+1). The modeled nucleotides are indicated with color-filled circles and the unmodeled nucleotides in empty circles. RPC2 residues that are involved in the binding of DNA–RNA hybrid are indicated and colored in gray. **b** Cryo-EM map of the human Pol III PTC in two different views. Critical regions are highlighted and encircled with dashed lines. The color scheme is used in the structure figures below if not elsewhere specified. **c** Structural model of the Pol III PTC. **d** Superimposed cryo-EM maps of the human Pol III PTC (colored as in Fig. 1b) and EC (gray) in this study. Conformational differences in the three modules are highlighted with dashed lines and arrows. Modular displacements are indicated with black arrows. **e** The map and model show unambiguous assignment of DNA–RNA hybrid in the PTC. The cryo-EM map is shown in the blue mesh.

The above results showed that the purified Pol III, the TS1^T5^, and TS1^T7^ templates were active and could be used in further biochemical and structural investigations. We next assembled human Pol III with TS1^T7^ to ensure that the majority of the complex exists in a PTC, instead of an EC state (Fig. 1a). In this scaffold, we placed the first deoxythymidine at position +2 (relative to the putative nucleotide addition site) so that all the deoxythymidines are exposed for effective binding to Pol III. The assembled PTC mimics the Pol III pausing on a stronger termination site of target genes.

The structure was determined using cryo-EM single-particle reconstruction. The cryo-EM 3D classification revealed two distinct conformational states, which represent a PTC and an EC, respectively. The two cryo-EM maps were both refined to nominal resolution at 3.6 Å (Fig. 1a, c, Table 1, Supplementary Fig. 2a–c, and Movies 1, 2). Focused refinement improved the cryo-EM map of the PTC core complex to a resolution of 3.3 Å (Table 1 and Supplementary Fig. 2a, d). The cryo-EM maps of Pol III EC and PTC enabled structural model building with our previously reported Pol III EC structure as a template (PDB: 7DU2)^21^. In the two structural models, all of the 17 subunits were traced with the majority of the residues unambiguously assigned (Supplementary Fig. 3). The majority of the DNA–RNA scaffold was assigned in both structural models. The single-stranded non-template strand was well-ordered in the PTC but invisible in the EC.

**Table 1:** Statistics of cryo-EM data collection, refinement, and validation.

	PTC	PTC core	EC
	(EMDB-31622) (PDB 7FJJ)	(EMDB-31622)	(EMDB-31621) (PDB 7FJI)
Data collection and processing			
Magnification	130,000x	130,000x	130,000x
Voltage (kV)	300	300	300
Electron exposure (e^–^/Å^2^)	50	50	50
Defocus range (μm)	−1.3 to −2.3	−1.3 to −2.3	−1.3 to −2.3
Pixel size (Å)	1.054	1.054	1.054
Symmetry imposed	C1	C1	C1
Initial particle images (no.)	1,771,522	1,771,522	1,771,522
Final particle images (no.)	48,593	131,442	40,590
Map resolution (Å)	3.6	3.3	3.6
FSC threshold	0.143	0.143	0.143
Map resolution range (Å)	3.3–8.5	3.3–5.5	3.1–8.5
Refinement			
Initial model used (PDB code)	7DU2		7DU2
Model resolution (Å)	3.6		3.6
FSC threshold	0.5		0.5
Map sharpening B factor (Å^2^)	−72.1	−77.2	−48.9
Model composition			
Non-hydrogen atoms	39,478		39,537
Protein residues	4847		4862
Nucleotide residues	48		43
Ligands	1× SF4, 7× Zn, 1× Mg		1× SF4, 7× Zn, 1× Mg
B factors (Å^2^)			
Protein	120.97		175.81
Nucleotide	164.32		211.75
Ligand	153.27		198.51
R.m.s deviations			
Bond lengths (Å)	0.006		0.007
Bond angles (°)	0.778		0.921
Validation			
MolProbity score	2.21		2.44
Clashscore	16.08		13.05
Poor rotamers (%)	0.05		0.58
Ramachandran plot			
Favored (%)	91.68		90.85
Allowed (%)	8.22		8.94
Disallowed (%)	0.11		0.21

### Overall structure of the PTC

The cryo-EM map of the PTC reveals the continuous density of the strand^NT^ and well-separated deoxythymidines (Fig. 1e), which extends from the unwinding site (transcription fork) and inserts into a strand^NT^ exit tunnel formed by the Pol III lobe and protrusion (Fig. 1b, c). Registration of the DNA–RNA hybrid within the EC and PTC was proposed according to the cryo-EM density around the paired DNA duplex and the design of the hybrid (Fig. 1a, e).

**Fig. 2:**
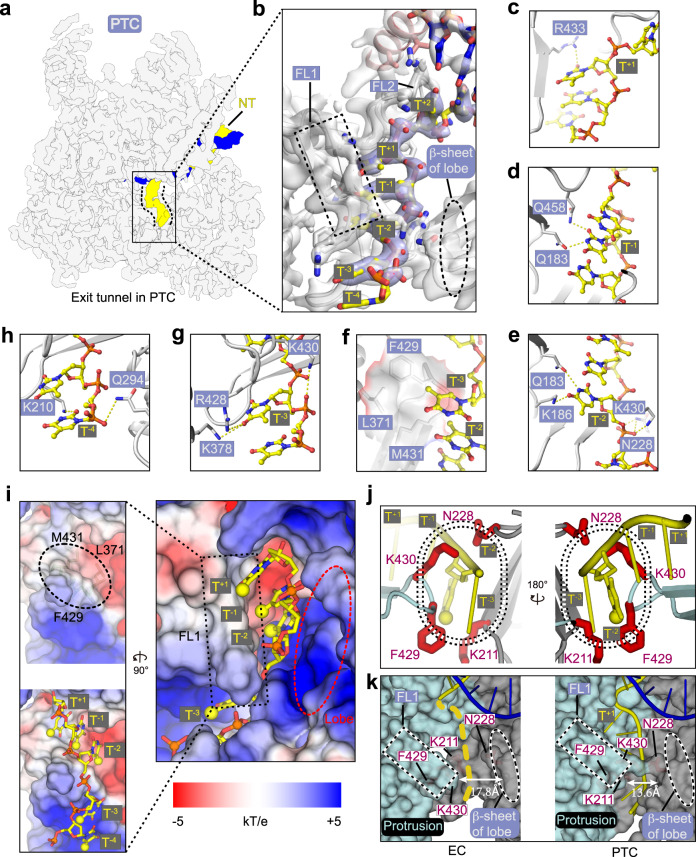
Binding of poly-dT within the exit tunnel. **a**, **b** The DNA–RNA hybrid and strand^NT^ exit tunnel within the PTC. Template strand (blue) and non-template strand (yellow) are highlights in the transparent cryo-EM map (**a**). Close-up view of the exit tunnel with the cryo-EM map shown in transparent surface and the structural model shown in ribbon and sticks (**b**). **c**–**h** Close-up views of interactions between Pol III and poly-dT (T^+1^ to T^−4^) around the exit tunnel. Residues involved in the interactions are shown in sticks. **i** Electrostatic potential surface of strand^NT^ exit tunnel in the PTC. The poly-dT is shown in sticks with the methyl groups highlighted in yellow balls and phosphate atoms in red balls. **j** Two-sides view of the hydrophobic gate. The gating residues and T^−2^ are shown in sticks and the methyl group of T^−2^ is shown in a yellow ball. **k** The close-up views of the exit tunnel in EC and PTC are shown on the surface in a similar orientation. The dashed yellow line indicates a putative non-template exit path in EC. The distance between the Cα atoms of protrusion residue R428 and lobe residue T229 is shown to indicate the width of the exit tunnel. The protrusion is colored in light cyan and the lobe is colored in gray.

The double-stranded DNA unwinds into the branched non-template and template strands over the bridge helix and the fork loop 2 (FL2) near the active site (Fig. 1e). The template strand undergoes a sharp kink at the +1 position and then forms a hybrid with the mimetic RNA transcript. From 3′ to 5′, the strand^NT^ (G^+18^ to C^+3^) is paired with the template strand, undergoes a sharp kink between T^+2^ and T^+1^, is trapped within the exit tunnel (T^+1^, T^−1^, and T^−2^), and makes a slight kink and flanks out of the exit tunnel (T^−3^ to T^−4^) (Figs. 1e, 2a, b).

### Structural comparison suggests modular reorganization from EC to PTC

**Fig. 3:**
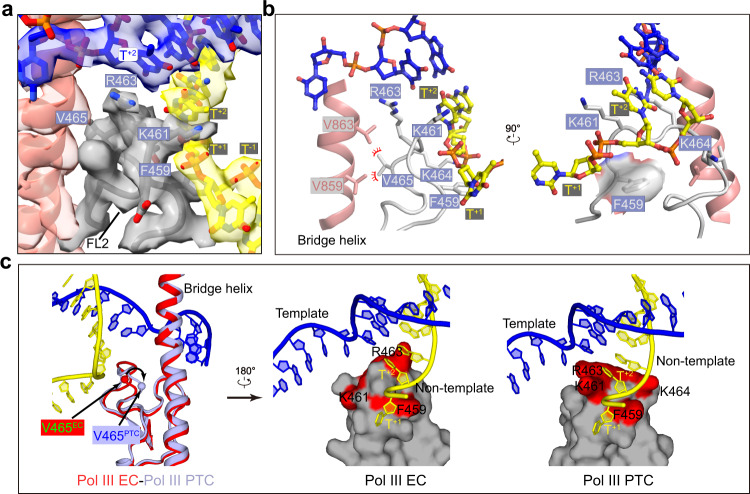
Structural differences of Pol III PTC and EC in the FL2 and transcription fork. **a** Close-up view of the locally refined cryo-EM map of the PTC. The map is shown on a transparent surface while the FL2 and transcription fork are shown in sticks. **b** Close-up views of contacts between the FL2 and non-template strand. Red dashes represent van der Waals contact. **c** Structural differences of the human Pol III EC and PTC in this study around the transcription fork. Superimposed structural models are shown in the left panel. The EC (middle) and PTC (right) are shown in a similar orientation for comparison. Nucleotides are shown in sticks and the DNA-contacting motif in the FL2 is shown on the surface.

**Fig. 4:**
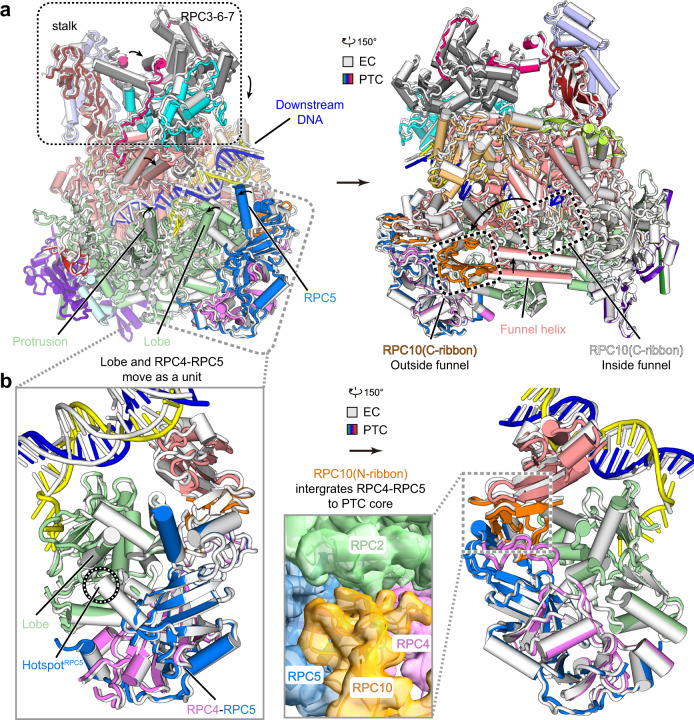
Structural differences of Pol III EC and PTC in the peripheral subcomplexes. **a** Structural comparison of the human Pol III EC (gray) and PTC (colored, similar to Fig. 1c). Structural differences are indicated with black arrows. **b** Close-up view of the structural difference around the RPC2 lobe. The locally refined cryo-EM map indicates that the N-ribbon of RPC10 binds RPC2 and supports the binding of RPC4-RPC5 to the PTC core.

The overall structure of PTC is generally similar to that of EC (Fig. 1c, d and Supplementary Movie 3)^20–22^, consistent with the previous findings that PTC is transcriptionally active^12^, albeit exhibiting the decreased elongation rate^35^. Structural comparison of the PTC and EC reveals a more compact core of PTC than that of EC and substantial conformational differences outside the core complex, suggesting the following modular reorganization during the transition from EC to PTC (Supplementary Movie 3). (I) Around the strand^NT^ exit tunnel, the RPC2 protrusion moves toward the active site, the lobe, and the associated RPC4-RPC5 moves toward the protrusion, generating a compact tunnel that partially grasps the poly-dT in the PTC (Fig. 2). (II) Around the transcription fork, the FL2 contacts and stabilizes the non-template and template strands and may prevent strand translocation (Fig. 3). (III) Around the Pol III funnel, the characteristic funnel helices move inwards, generating a narrower funnel in the PTC (Fig. 4). Consistently, the C-terminal ribbon domain (C-ribbon) of RPC10 (Fig. 4a and Supplementary Fig. 4a) was observed in the funnel in the cryo-EM map of EC^20–22^, but not in that of PTC (Supplementary Fig. 4b). Additionally, we observed minor movement of a clamp and associated RPC3-RPC6-RPC7 toward DNA, resulting in a slightly narrower cleft in the PTC (Fig. 4a). The above structural differences reflect distinct conformational states of Pol III resulted from the trapped (in PTC) and free (in EC) poly-dT in the strand^NT^ exit tunnel. The poly-dT and exit tunnel in the PTC conformation were not observed in previously reported Pol III EC structures^20–22^, suggesting a poly-dT-dependent transition from EC to PTC for transcription termination.

### Poly-dT inserts into and is well-stabilized by the exit tunnel in the PTC

The cryo-EM map at near-atomic resolution reveals well-ordered strand^NT^ winding through the exit tunnel, which is formed by the four-stranded β-sheet of the RPC2 lobe (residues 186–236) on one side, the fork loop 1 (FL1) on the other side, and a part of the protrusion on the bottom (Fig. 2a, b). Three deoxythymidine nucleotides, T^−2^, T^−1^, and T^+1^ of the strand^NT^ are trapped within the exit tunnel through a network of hydrogen bonds and hydrophobic interactions. The hydrophobic side of poly-dT (multiple methyl groups) faces toward the hydrophobic surface of the protrusion whereas the acidic side of poly-dT (multiple phosphate groups) faces toward the positively charged surface of the lobe (Fig. 2i). The complementary contacts between poly-dT and the exit tunnel suggest a specific binding of the exit tunnel to the poly-dT, but not other motifs.

The base of T^+1^ is in proximity to the side chains of FL1 residue R433 (Fig. 2c). The T^−1^ makes hydrogen bonds with side chains of FL2 residue Q458 and lobe residue Q183 (Fig. 2d). The phosphate group of T^−2^ is stabilized by FL1 residue K430 and lobe residue N228 and the base of T^−2^ is stabilized by the side chains of lobe residues Q183 and K186 (Fig. 2e). Hydrophobic residues F429, L371, and side chain of K211 resemble a hydrophobic gate that separates the bases of T^−3^ and T^−2^, generating a slight kink of the strand^NT^ (Fig. 2b, f, i–k). The methyl group of T^−2^ faces toward the hydrophobic gate, suggesting that translocation of tandem deoxythymidine nucleotides through this hydrophobic gate might be energetically unfavorable and lead to the pause of Pol III on DNA (Supplementary Movie 3).

Outside the tunnel, T^−3^, T^−4^, and the 3′ flanking region are exposed and extend outward (Fig. 2b). The base of T^−3^ makes hydrogen bonds with FL1 residue R428 and K378 of the protrusion helix and the phosphate group of T^−3^ is stabilized by the side chain of FL1 residue K430 (Fig. 2g). The base and phosphate group of T^−4^ contact side chains of lobe residues K210 and Q294, respectively (Fig. 2h), consistent with a previous study showing that termination-defect mutations are mapped to this region^36^. The map around T^−4^ is weak, indicative of its high flexibility (Fig. 2b). The upstream non-template strand is invisible due to its flexibility and would form upstream DNA duplex with the template strand. The abovementioned poly-dT-contacting residues are highly conserved across species (Supplementary Fig. 5), suggesting a conserved mechanism.

Structural comparison of Pol III EC and PTC shows well-ordered strand^NT^ within a narrow exit tunnel in the PTC and an open exit tunnel in the EC (Fig. 2k, Supplementary Fig. 4b, and Movie 3). In contrast to that in the PTC, the strand^NT^ is highly flexible and could not be evidently observed in cryo-EM maps of the EC from our study and others^20–22,26^. Consistently, the protrusion and the lobe are separated by ~14 Å in the PTC but ~18 Å in the EC. Notably, residues K430 of the FL1 and N228 of the lobe in the PTC spans and binds the phosphate backbone between T^−2^ and T^−3^ (Fig. 2e, i, k, right panel). No such interactions were observed in the EC (Fig. 2k, left panel). The above structural differences suggest an induced-fit binding of poly-dT to the exit tunnel during the transition from EC to PTC.

### The fork loop 2 stabilizes the transcription fork and prevents strand translocation

The cryo-EM map of the PTC shows a well-ordered transcription fork, bridge helix, and FL2 (residues 456 to 473 of RPC2) (Fig. 3a). The FL2 inserts into the transcription fork and strand separation appear to take place at +2, where the base-pair is more separated compared to downstream dsDNA (Fig. 1e). A DNA-contacting motif (F^459^DKTRKV^465^) of FL2 is in parallel with the phosphate backbone around T^+2^ and guides the strand^NT^ to run toward the exit tunnel. Residue R463 stacks with the base of T^+2^ of the template strand. Residue K461 stacks with the base of T^+2^ of the strand^NT^. Residue F459 under the T^+2^ supports the ribose groups of T^+2^ and T^+1^. The strand^NT^ makes a sharp kink over residue F459 by ~90° with the upstream region running toward the exit tunnel (Figs. 1e, 2b, 3b). Thus, the highly conserved DNA-contacting motif (Supplementary Fig. 5) of the FL2 stabilizes both template and non-template strands at the transcription fork and may prevent strand translocation.

Structural comparison of Pol III EC and PTC shows a conformational difference in FL2 (Fig. 3c, left panel), especially at the DNA-contacting motif. The FL2 in Pol III PTC adopts an open state^37^ (Supplementary Fig. 6a) and appears to be stabilized by residue V465, which makes hydrophobic contacts with residues V859 and V863 of the bridge helix (Fig. 3b, c, left panel and Supplementary Movie 3). In contrast, the DNA-contacting motif tends to be flexible in Pol III EC (Supplementary Fig. 6a) and V465 is positioned away from the bridge helix (Fig. 3c and Supplementary Movie 3), possibly due to the lack of stable contact with strand^NT^. The disruption of this contact leads to less stabilized FL2 in Pol III EC, as evidenced by the weak cryo-EM map of FL2^21,22^. Rearrangement of FL2 from EC to PTC may result from the stabilization of poly-dT in the PTC and in turn, prevent strand translocation near the transcription fork (Fig. 3c and Supplementary Movie 3).

Previous studies had shown that several mutations in RPC2 can affect the process of termination. Termination-altering mutations were mapped to residues 300–325 and 455–521 of yeast RPC2^17,36^. The first region is highly variable while the second one is highly conserved. The human RPC2 region 282–311 (300–325 in yeast RPC2) is located proximal to and makes contact with the 5′ terminus of poly-dT (Fig. 2h). Residues 437–503 (455–521 in yeast RPC2) are in proximity to the transcription fork and cover the sequence of FL2. This region may involve the stabilization of the transcription fork and prevents strand translocation (Fig. 3a, b), consistent with the critical role of the FL2-containing region in transcription termination^36^.

### A potential role of termination-reinitiation subcomplex in transcription termination

In yeast, the termination-reinitiation subcomplex C53-C37-C11 (the equivalent of the human RPC4-RPC5-RPC10) is believed to regulate Pol III transcription termination. The Pol III devoid of this subcomplex has no decrease in transcript output but requires more dTs in termination, indicative of a deficient termination^30,31^. It has been reported that a similar termination defect may result from the deletion of a C-terminal region (residues 226–230) of C37 (RPC5 counterpart), which was termed a hotspot in decreased termination mutants in vivo^38^.

The Pol III EC structures show that the lobe directly binds RPC4-RPC5 on the hotspot^20–22^ (Fig. 4a and Supplementary Fig. 4d), suggesting that RPC4-RPC5 may involve in termination through stabilizing the associated lobe of the exit tunnel. The lack of RPC4-PRC5 would allow the lobe to be more flexible and tend to be positioned away from the exit tunnel, making the poly-dT-induced binding unfavorable. The deletion of the hotspot may lead to similar destabilization of the lobe.

It is known that RPC10 is essential for the termination but is independent of its RNA cleavage activity^32^. Similar to that in Pol III EC structures^20–22^, the N-terminal ribbon domain (N-ribbon) of RPC10 in the PTC structure binds the polymerase core and may support binding of RPC4-RPC5 to the core enzyme (Fig. 4b). The C-ribbon of RPC10 is homologous to the Pol II elongation factor TFIIS^35^ and is flipped out of the funnel in the PTC (Fig. 4a and Supplementary Fig. 4b). Previous studies showed that the depletion of RPC10 leads to the dissociation of the termination-reinitiation subcomplex^30^ and that adding back of recombination C53-C37 to the purified core enzyme restores termination in the absence of C11^31,39^. The above results collectively suggest that RPC10 involves transcription termination through maintaining the association of C53-C37 (RPC4-RPC5) with the polymerase.

### Structural comparison with Pol I and Pol II reveals a Pol III-specific termination mechanism

**Fig. 5:**
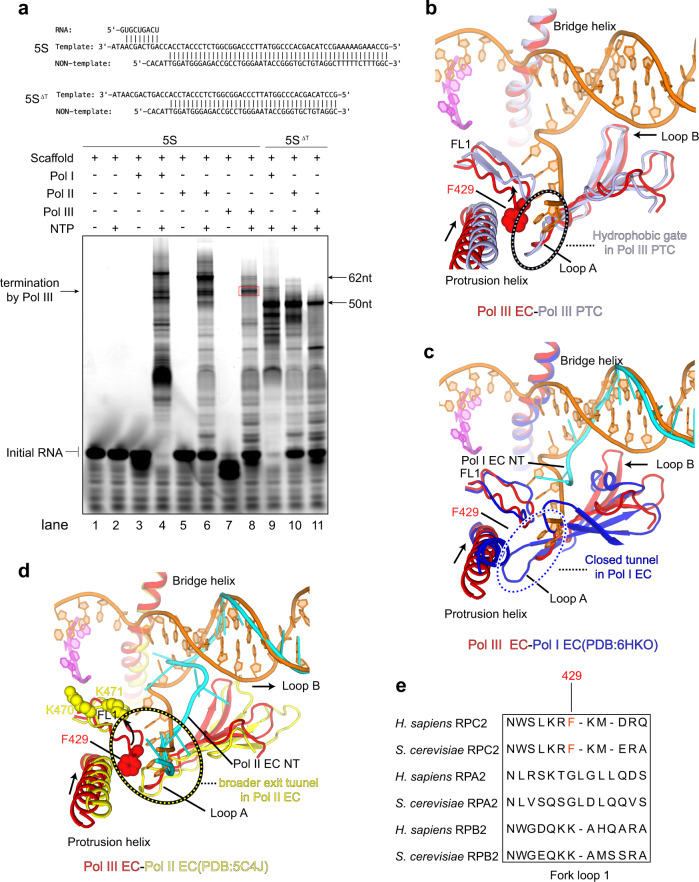
Comparison of the exit tunnel of Pol III EC/PTC and counterparts of Pol I and Pol II EC. **a** Schematic model of human 5S rDNA scaffolds used in RNA extension assays. The RNA primer is 5′-FAM-labeled for product detection. 5S: scaffold with strong poly-dT termination signal; 5S^ΔT^: scaffold lacking poly-dT termination signal and indicating the minimum length of Pol III termination products. Same amount of Pol I, Pol II, and Pol III were incubated with scaffolds on ice for 30 min, respectively. 1.25 mM NTPs (ATP, CTP, GTP, UTP) were added and reactions were started at 37° for 30 min. RNA products were detected using the 5′-FAM fluorescence label on RNA primers. A red box indicates specific termination by human Pol III. Results are representative of at least three independent experiments. **b**–**d** Close-up views of the superimposed human Pol III EC (red) with the human PTC in this study (gray), **b** the yeast Pol I EC (PDB: 6HKO, blue), **c** the yeast Pol II EC (PDB: 5C4J, yellow), **d** Dashed circles indicate the exit tunnel in Pol III PTC, the closed tunnel in Pol I EC, and broader tunnel in Pol II EC. Residue F429 in human Pol III EC and equivalent residues K470/K471 in yeast Pol II EC are shown in spheres to highlight the conformational difference. DNA and the exit tunnel are shown and the rest parts are omitted for clarity. Structural differences are indicated with arrows. **e** Multiple sequence alignment of the FL1 of the three eukaryotic RNA polymerase (Pol I, II, III from *Homo sapiens* and *Saccharomyces cerevisiae*). The highlighted (in red) Pol III-specific residue, F429, is critical for the formation of the exit tunnel in Pol III.

We next performed in vitro transcription elongation assay using DNA fragments derived from human 5S rDNA, a natural Pol III-transcribed gene, which has a strong termination signal (TTTTTCTTT) in the non-template strand (Supplementary Table 1). As positive controls (Fig. 5a, lanes 9, 10, and 11), Pol I, Pol II, and Pol III generated transcription products with comparable size (length of RNA products) on the poly-dT-lacking scaffold (5S^ΔT^ scaffold). Pol I and Pol II could read-through 5S rDNA scaffold (Fig. 5a, lanes 4 and 6) whereas Pol III efficiently terminated at a specific region (Fig. 5a, lanes 4, 6, and 8), supporting a critical role of poly-dT specifically in Pol III-mediated transcription termination.

To investigate why poly-dT-mediated termination specifically occurs in Pol III but not in Pol I or Pol II, we compared the structures of human Pol III PTC/EC (Fig. 5b) with yeast Pol I EC (PDB: 6HKO)^40^ and yeast Pol II EC (PDB: 5C4J)^37^, which both contain visible nucleotides in equivalent strand^NT^ tunnels. Compared to Pol III EC/PTC structures, the Pol I EC structure^40^ reveals a nearly closed tunnel (equivalent to the hydrophobic gate in Pol III PTC) through the interaction between the counterparts of the FL1 and a loop of the lobe (Fig. 5c and Supplementary Fig. 6b). The observed strand^NT^ is positioned above the equivalent exit tunnel and the hydrophobic gate, suggesting that no nucleotide could be trapped in the sealed tunnel in Pol I. Consistent with the structural differences, the termination mediated by mammalian Pol I requires two *trans*-regulatory factors, TTF-1 and PTRF, which specifically recognize the two *cis*-regulatory elements, the Sal box and T-rich sequence^41^.

The crystal structure of yeast Pol II EC^37^ reveals a well-ordered strand^NT^ in an exit tunnel similar to that in Pol III and extends further to pair with the upstream template strand (Fig. 5d and Supplementary Fig. 6c). Instead of being stabilized near the protrusion helix in Pol III EC/PTC, the FL1 equivalent in Pol II is positioned near the fork of the upstream duplex, leading to a broader exit tunnel^37^. Cryo-EM structure of mammalian Pol II EC (PDB: 5FLM)^42^ and our recently reported Pol II preinitiation complex structure (PDB: 7EG7)^33^ also reveal similar conformation of FL1 to that in the yeast Pol II EC. In agreement with the structural difference, sequence alignment of FL1 (Fig. 5e) shows that the critical residue (F429) of human Pol III in trapping poly-dT is converted to lysine in Pol II. Consistently, the termination of Pol II-mediated transcription is coupled with the mRNA 3′-end processing^7^ and controlled by multiple factors but is independent of the template sequence.

## Discussion

The strand^NT^ exit tunnel adopts an open conformation in the EC but a closed conformation in trapping of the strand^NT^ in the PTC, suggesting a working model to elucidate how Pol III efficiently mediates transcription elongation throughout target genes and specifically terminates at poly-dT. (I) In Pol III-mediated transcript elongation, regular strand^NT^ has no specific contact with the exit tunnel and easily passes through the open exit tunnel, permitting an efficient strand translocation. (II) The poly-dT of the strand^NT^ enters the exit tunnel and tends to generate specific intermolecular contacts and induce closure of the exit tunnel, which in turn stabilizes and traps the strand^NT^. (III) The methyl groups of the poly-dT face toward the partially closed hydrophobic gate, possibly generating hydrophobic repulsion and a higher energy barrier to prevent the translocation of the strand^NT^. (IV) The FL2 stabilizes the transcription fork and may prevent the translocation of both non-template and template strands. As a result, the Pol III-mediated transcription elongation tends to be paused at the poly-dT region and the DNA retention of Pol III may correlate with the number of tandem deoxythymidines (Supplementary Movie 3). Sufficient tandem deoxythymidines would collectively decrease the rate of strand^NT^ translocation to a threshold that is required for termination.

We assembled the memetic PTC in an in vitro system using DNA–RNA hybrid in the presence of poly-dT in the strand^NT^. In the final reconstruction, ~40 K particles were used for the refinement of EC and ~48 K particles for PTC (Supplementary Fig. 2a). Consistently, read-through by Pol III was observed in transcription on TS1^T5^ and TS1^T7^ templates. These observations suggest a dynamic equilibration between EC and PTC, which may be required for efficient scanning of poly-dT during the transition from EC to PTC or the release of polymerase from poly-dT after generation of PTC. It is also possible that other uncharacterized factor(s) may facilitate the EC to PTC conversion and/or efficiency of termination in cells.

The retention of Pol III on poly-dT may lead to the dissociation of polymerase and RNA products from the genome with the mechanism not fully understood. A previous study showed that the destruction of Pol III is essential for the release of RNA^13^. Despite considerable changes in the exit tunnel and associated regions, the formation of PTC unlikely affects the polymerase stability. The core region of PTC remains largely unchanged when compared to the cryo-EM structure of the human Pol III EC, with a root-mean-square deviation (RMSD) of 0.74 Å for 1881 Cα atoms (Fig. 4a).

It has been proposed that the generated rU:dA hybrid at Pol III active site is unstable and the RNA product tends to dissociate from the PTC, possibly facilitating the release of RNA and transcription termination^32^. Distinct from Pol III, Pol I and Pol II use multipartite *cis*-regulatory elements and *trans*-acting factors to terminate the transcription, as indicated in the previously proposed torpedo model^1,7^. Intriguingly, bacterial RNA polymerase uses factor-dependent and intrinsic mechanisms to terminate transcription^5,6,43^ and the intrinsic termination requires a stretch of poly-dT. Distinct from that in Pol III, the bacterial RNA polymerase terminates transcription through generating an RNA hairpin structure immediately followed by an oligo(rU) sequence in the nascent RNA.

The yeast “termination-reinitiation” subcomplex C53-C37-C11 (the equivalent of human RPC4-RPC5-RPC10) can be dissociated from Pol III during purification from an *S. cerevisiae* C11 mutant. The resulted core polymerase has no decrease in transcript output but requires more dT (8–9 dTs) to terminate transcription^30^. Comparison of our human Pol III EC and PTC structure suggests that RPC4-RPC5 and N-ribbon of RPC10 directly interact with the RPC2 lobe. By stabilizing the RPC2 lobe, RPC4-5 may indirectly facilitate trapping poly-dT effectively. In the PTC map, we have not observed any additional density of the RPC5 C-terminal domain that is specific to mammals. This indicates that the human-specific C-terminal domain of RPC5 may have no direct role in trapping of poly-dT although we can’t fully exclude this possibility. During the transition from EC to PTC, the unchanged position of this subcomplex to RPC2 lobe indicates that the “termination-reinitiation” subcomplex may play a regulatory role in the formation of the non-template exit tunnel. Depletion of the “termination-reinitiation” subcomplex may destabilize the lobe and impair the termination gate formation. Thus, more tandem deoxythymidines are required for termination. An N-terminal loop adjacent to the hotspot of RPC5 is referred to as the “termination-reinitiation” loop and may play an important role during the transition from termination to reinitiation, the mechanism of which requires further investigation.

## Methods

### Protein purification, in vitro RNA extension assays, and complex assembly

Human Pol I was purified from transient-transfected HEK Expi293F cells as indicated in our recent report^44^. Mammalian Pol II was isolated from pig (*S. scrofa*) thymus as indicated in our recent studies^33,34^. Four residue differences (G882S of RBP2, T75I of RPB3, S140N of RPB3, and S126T of RPB6) exist between *S. scrofa* and *H. sapiens* Pol II^33,34^. Protein purification of human Pol III was performed as previously indicated^21^.

RNA extension assays were performed to test the termination activity of polymerases on human 5S rDNA and variants based on scaffold used for structural investigation in this study. A short 5′-FAM-labeled RNA primer 5′-FAM-GUGCUGACU was used for fluorescence detection in all assays. The DNA sequences used for RNA extension assays are listed in Supplementary Table 1. Template DNA and non-template DNA were suspended in DNA folding buffer containing 20 mM HEPES pH 8.0, 100 mM NaCl, and annealed by first incubating at 95 °C for 10 min and decreasing the temperature from 95 to 20 °C at a rate of 1 °C/min. To obtain dsDNA–RNA hybrid, DNA duplex was incubated with a 1.2-fold excess of RNA for 10 min at 40 °C and then cooled to 4 °C. Briefly, the transcription reactions contained 500 nM dsDNA–RNA hybrid, 600 nM polymerase, 40 mM NaCl, 60 mM KCl, 20 mM Na-HEPES pH 8.0, 2 mM MgCl_2_, 2 mM DTT, and 1.25 mM NTPs (ATP, CTP, GTP, and UTP). Polymerase was first assembled with dsDNA–RNA hybrid on ice for 30 min. 4× assay buffer and 4× NTP solution were added to the polymerase-scaffold complex to initialize the reaction. Transcription assays were performed at 37 °C for 30 min and stopped by a 2× quenching buffer (1× TBE buffer, 20 mM EDTA pH 8.0, and 8 M Urea) in a 1:1 ratio. The RNA products were analyzed on 10% acrylamide-urea gels. About 4 μl sample was loaded to each lane. The gels were run with a power of 15 W for 70 min in 0.5 × TBE buffer. RNA signal was detected by scanning the fluorescence of the 5′-FAM label on the RNA primer.

The PTC was assembled essentially similar to our previously reported reconstitution of human Pol III EC^21^. Given that PTC is transcriptionally active, to capture the pretermination state, we designed an elongation scaffold that contains 12 mismatched nucleotides and seven deoxythymidines in the non-template strand. Template DNA: 5′-GAAACAGCGGCTCTAGTCTGTCCAGCACCCGTAGCACCCGGTATTCCCAGGCGG-3′, non-template DNA: 5′-CCGCCTGGGAATACCGGGTGCTTACGATTTTTTTCAGACTAGAGCCGCTGTTTC-3′, and RNA: 5′-CCGGGUGCUG-3′. The Pol III PTC complex was incubated with fresh prepared DNA–RNA scaffold on ice for 30 min at a 1:2 molar ratio. The sample was dialyzed in buffer containing 20 mM HEPES, pH 8.0, 150 mM NaCl, 2 mM MgCl_2_, and 2 mM DTT at 4 °C for 12 h using Slide-a-lyzer mini dialysis pins (10,000 MW cut-off, Thermo Fisher).

### Cryo-EM sample preparation

For negative staining EM grids preparation, 5 μL of Pol III PTC complex sample were applied onto glow-discharged copper grids supported by a continuous thin layer of carbon film for 60 s before negatively stained by 2% (w/v) uranyl formate solution at room temperature. The grids were prepared in the Ar/O_2_ mixture for 15 s using a Gatan 950 Solarus plasma cleaning system with a power of 15 W. The negatively stained grids were loaded onto a Thermo Fisher Scientific Talos L120C microscope equipped with a Ceta CCD camera and operating at 120 kV at a nominal magnification of 92,000x, corresponding to a pixel size of 1.58 Å on the specimen.

For cryo-EM grids preparation, 3 μL of the sample at a concentration of 0.7 mg/mL dialyzed PTC complexes were applied to freshly glow-discharged Quantifoil R 1.2/1.3 holey carbon grids. After incubation of 5 s at a temperature of 4 °C and a humidity of 100 %, the grids were blotted for 8.5 s in a Thermo Fisher Scientific Vitrobot Mark IV and plunge-frozen in liquid ethane at liquid nitrogen temperature. The grids were prepared in the H_2_/O_2_ mixture for 30 s using a Gatan 950 Solarus plasma cleaning system with a power of 5 W. The ø 55/20 mm blotting paper (TED PELLA) is used for plunge freezing.

### Data collection

The cryo-EM grids of PTC were loaded onto a Thermo Fisher Scientific Titan Krios transmission electron microscope equipped with a Gatan GIF Quantum energy filter (slit width 20 eV) and operating at 300 kV for data collection. All the cryo-EM images were automatically recorded by a post-GIF Gatan K2 Summit direct electron detector in the super-resolution counting mode using Serial-EM^45^ with a nominal magnification of 130,000x in the EFTEM mode, which yielded a super-resolution pixel size of 0.527 Å on the image plane, and with a defocus ranged from −1.3 to −2.3 μm. Each micrograph stack was dose-fractionated to 36 frames with a total electron dose of ~50 e^−^/Å^2^ and a total exposure time of 8.2 s. About 3125 micrographs from a total of 3400 micrographs were selected for further processing.

### Image processing and model building

For cryo-EM data, drift and beam-induced motion correction were applied on the super-resolution movie stacks using MotionCor2^45^ and binned twofold to a calibrated pixel size of 1.054 Å/pix. The defocus values were estimated by Gctf^46^ from summed images without dose weighting. Other procedures of cryo-EM data processing were performed within RELION v3.0 and 3.1^47^ and cryoSPARC v2^48^ using the dose-weighted micrographs.

A subset of ~10,000 particles were auto-picked free of reference and subjected to reference-free 2D classification. Some of the resulting 2D class averages were low-pass filtered to 20 Å and used as templates for automatic particle picking of the whole dataset in RELION v3.0 resulting in an initial set of 1,771,522 particles for several rounds of 2D and 3D classification. About 848,562 particles were selected from good classes for further 3D classification. After two rounds of 3D classification, 148,433 particles were subjected to a masked 3D classification for the whole particle without alignment. Two classes showed distinct features of single-stranded DNA between Protrusion and lobe were subjected to 3D homogeneous refinement in cryoSPARC, respectively. One class (48,593 particles) yielded a reconstruction (PTC) with a nominal global resolution of 3.6 Å. This map shows the continuous density of single-stranded DNA, including termination signal residues, extended from the unwinding site (transcription fork) to the junction position, while RPC10 is an outside funnel. The rest of the class (40,590 particles) was reconstructed using the same strategies and yielded a reconstruction (EC) with a nominal global resolution of 3.6 Å. In this map, there is no apparent density of single-stranded DNA in the same position compared with that in PTC. Interestingly, there is a stronger signal belonging to the RPC10 C-ribbon inside the funnel. To improve the map quality in the core region of PTC, a local mask 3D classification and a masked local refinement were performed. This refinement excludes the stalk module and trimer module due to intrinsic mobility. About 131,442 particles in one of the selected three classes were selected and subjected to 3D auto-refinement, CTF refinement, Bayesian polishing, and post-processing in RELION v3.1 yielding a reconstruction of the core region (PTC core) at 3.3 Å resolution.

All of the reported resolutions were based on the gold-standard Fourier shell correlation (FSC) = 0.143 criterion. The FSC curves were corrected for the effects of a soft mask with high-resolution noise substitution. All cryo-EM maps were sharpened by applying a negative B-factor estimated in cryoSPARC or RELION. Directional FSC curves and map anisotropy were assessed using the 3DFSC (https://3dfsc.salk.edu)^49^. All the visualization and evaluation of the 3D volume map and mask creation were performed within UCSF Chimera^50^. The local resolution variations were calculated in cryoSPARC v2 for PTC and EC and in RELION v3.1 for PTC core.

The structure of human Pol III EC (PDB: 7DU2) was used as starting structural template, which was docked into PTC core map by rigid-body fitting by UCSF Chimera. The model was then manually adjusted in COOT^51^. The resulting model was placed in the PTC map and followed by the fitting of rigid body groups in COOT. Groups for rigid body refinement were chosen on the basis of visual inspection of the fit to density. To build a structural model of EC, the PTC model was docked into the EC map by rigid-body fitting by UCSF Chimera and followed by manual adjustment in COOT. To improve model geometry, the PTC and EC models were subjected to real-space refinement in PHENIX^52^ with secondary structure and geometry restraints to prevent overfitting, respectively. The final models were validated using Molprobity^53^ and the FSC of the final models versus the maps, respectively. Statistics of the map reconstruction and model refinement can be found in Table 1. Figures for the structural model and the EM density map were prepared by PyMOL (https://pymol.org/) and UCSF ChimeraX^54^.

### Reporting Summary

Further information on research design is available in the Nature Research Reporting Summary linked to this article.

## Supplementary information

Supplementary Information

Description of Additional Supplementary Files

Supplementary Movie 1

Supplementary Movie 2

Supplementary Movie 3

Reporting summary

## Data Availability

The cryo-EM maps have been deposited in the EM Databank under accession numbers: EMD-31621 (elongation state) and EMD-31622 (pretermination state). Atomic coordinates have been deposited in the Protein Data Bank (http://www.rcsb.org/pdb) with PDB IDs: 7FJI (elongation state) and 7FJJ (pre-termination state). Source data are provided with this paper.

## Source data

Source Data
