# Supplementary material for: Structural insights into RNA polymerase III-mediated transcription termination through trapping poly-deoxythymidine
Source: Nat Commun. 2021 Oct 21;12:6135. doi: 10.1038/s41467-021-26402-9 (PMC8531034; doi:10.1038/s41467-021-26402-9)
Supplement: Supplementary file 1 — Supplementary Information [file 41467_2021_26402_MOESM1_ESM.pdf]

Supplementary Information For

Structural insights into RNA Polymerase III-mediated transcription termination through trapping poly-deoxythymidine

Haifeng Hou[1#], Yan Li[1#], Mo Wang[1#], Aijun Liu[1#], Zishuo Yu[1], Ke Chen[1], Dan Zhao[1], and Yanhui Xu[1,2,3,4]*

[1]Fudan University Shanghai Cancer Center, Institutes of Biomedical Sciences, State Key Laboratory of Genetic Engineering, Shanghai Key Laboratory of Radiation Oncology, and Shanghai Key Laboratory of Medical Epigenetics, Shanghai Medical College of Fudan University, Shanghai 200032, China.

[2]The International Co-laboratory of Medical Epigenetics and Metabolism, Ministry of Science and Technology, China, Department of Systems Biology for Medicine, School of Basic Medical Sciences, Shanghai Medical College of Fudan University, Shanghai 200032, China.

[3]Human Phenome Institute, Collaborative Innovation Center of Genetics and Development, School of Life Sciences, Fudan University, Shanghai 200433, China

[4]State Key Laboratory of Reproductive Regulation and Breeding of Grassland Livestock School of Life Sciences, Inner Mongolia University, Hohhot, P.R.China 010070

[#] These authors contributed equally to this work.

* To whom correspondence should be addressed. E-mail: xuyh@fudan.edu.cn

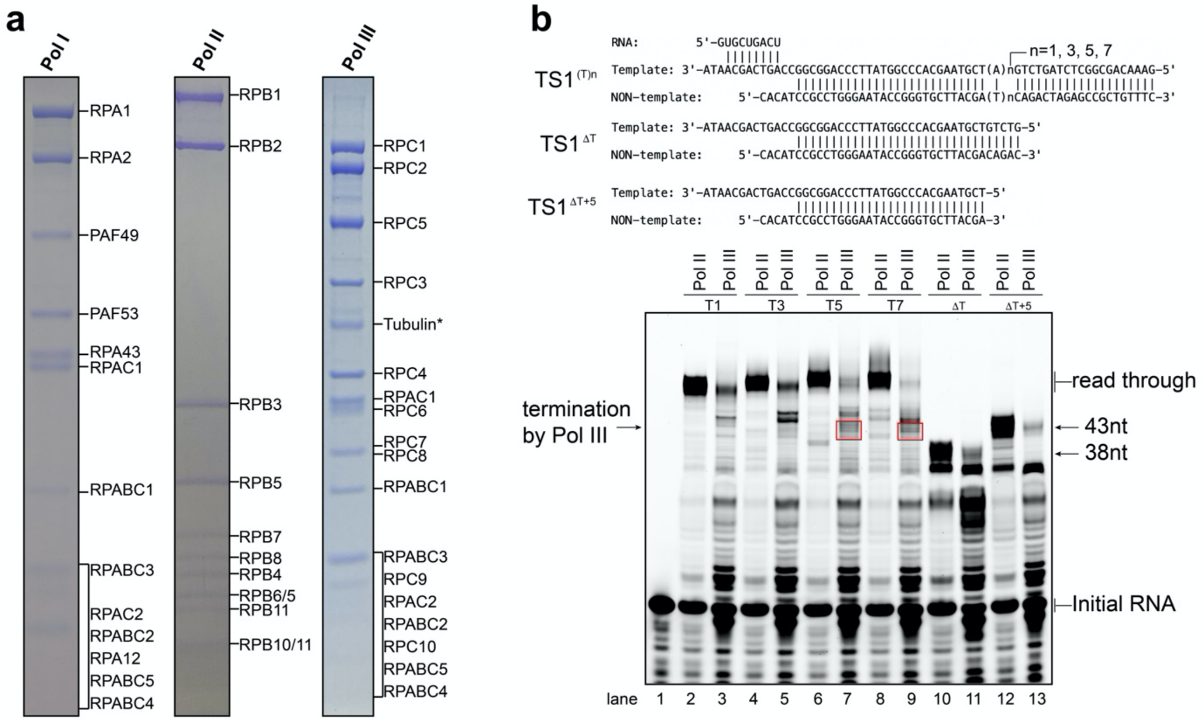

Supplementary Figure 1. Protein purification and RNA extension activity of human Pol III

(a) The purified recombinant human Pol I, Pol III and endogenous pig Pol II were subjected to SDS-PAGE followed by Coomassie blue staining. (b) Schematic model of poly-dT scaffolds used in RNA extension assays. The RNA primer is 5'-FAM labeled for product detection. $TS1^{(T)n}$: four scaffolds with internal poly-dT (n=1, 3, 5, 7); $TS1^{DT}$ indicates the minimum length of Pol III termination products; $TS1^{DT+5}$ indicates the potential Pol III termination site. Same amount of Pol II and Pol III were incubated with scaffolds on ice for 30min, respectively. 1.25mM NTPs (ATP, CTP, GTP, UTP) were added and the reactions were started at 37 degree for 30 min. Extended RNA products were detected using the 5'-FAM fluorescence label on RNA primers. Red boxes indicate specific termination by human Pol III. The experiment was repeated at least three times.

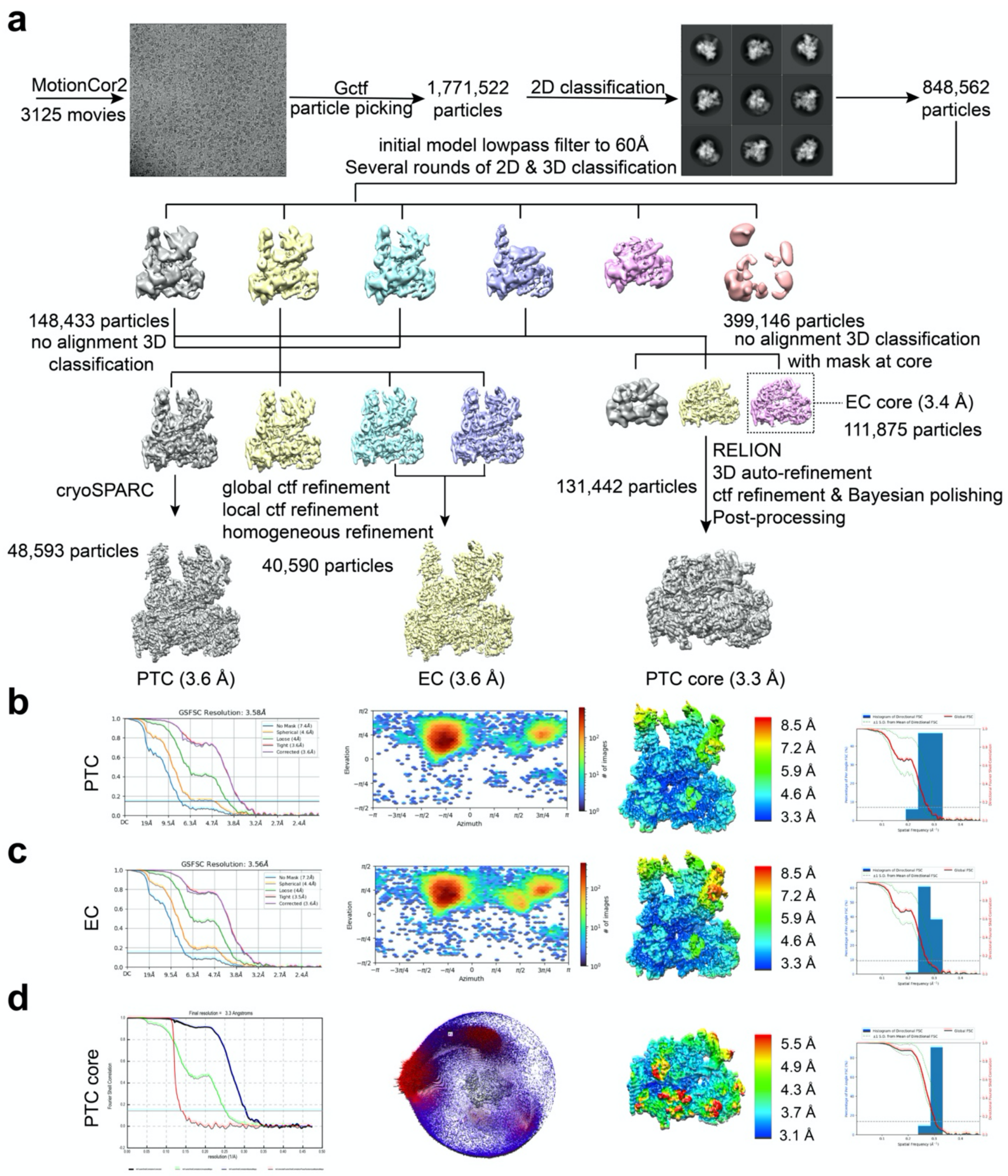

Supplementary Figure 2. Data collection and image processing.

(a) Flow-charts of the cryo-EM image processing and 3D reconstructions of the human Pol III PTC. (b-d) FSC curves, orientation of the cryo-EM reconstructions and local resolution estimation

of PTC, EC and PTC core, respectively. Directional FSC plots calculated by 3DFSC indicate the mostly isotropic maps without preferred orientation bias.

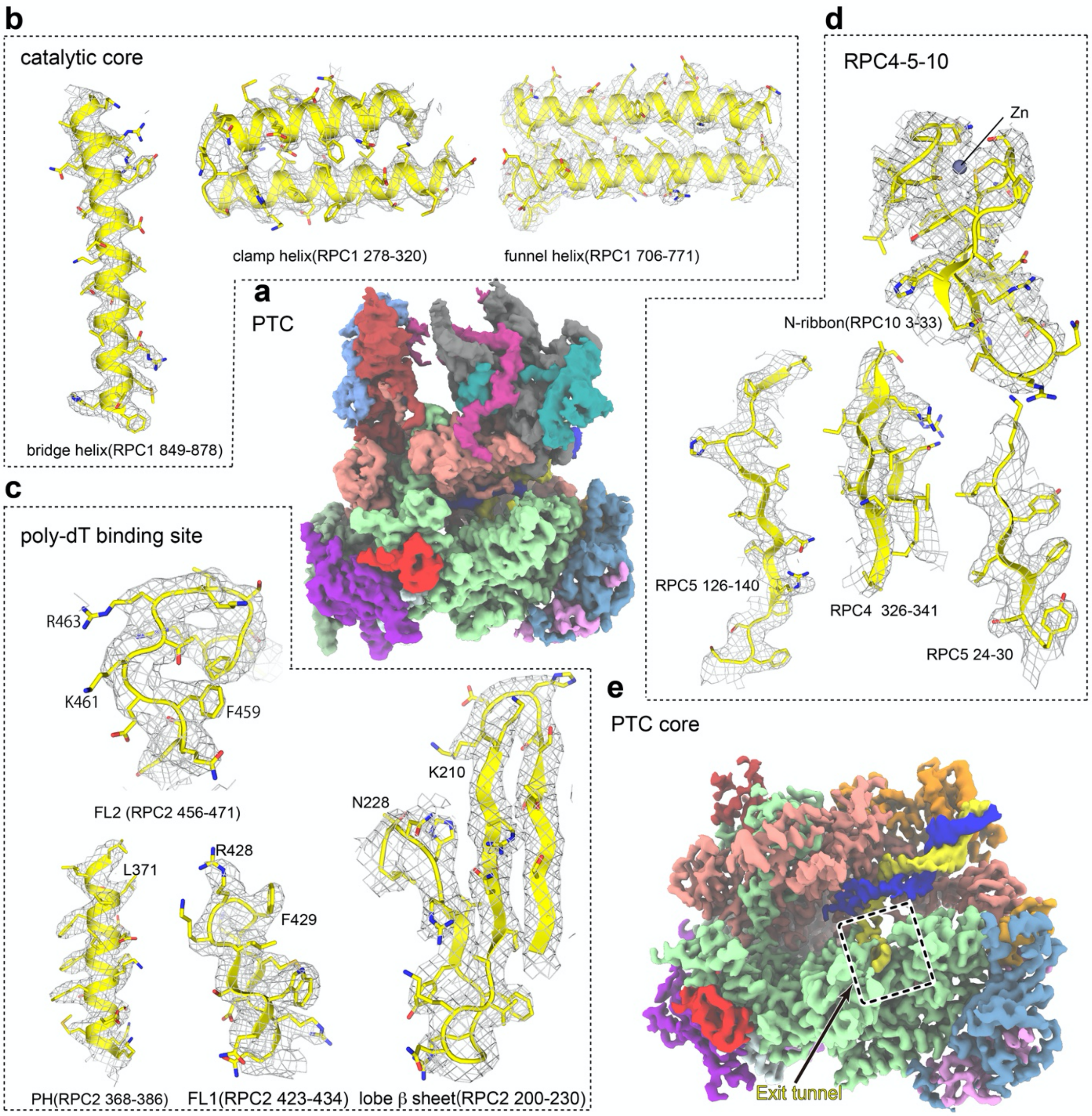

Supplementary Figure 3.Representative cryo-EM maps and structural models of the human Pol III PTC.

(a) Overall cryo-EM map of the human Pol III PTC shown as in Fig. 1. (b-d) Cryo-EM maps and structural models of representative regions. Most side chains fit in corresponding cryo-EM map, indicating the structure was correctly built. (e) Locally refined cryo-EM map in core region of PTC.

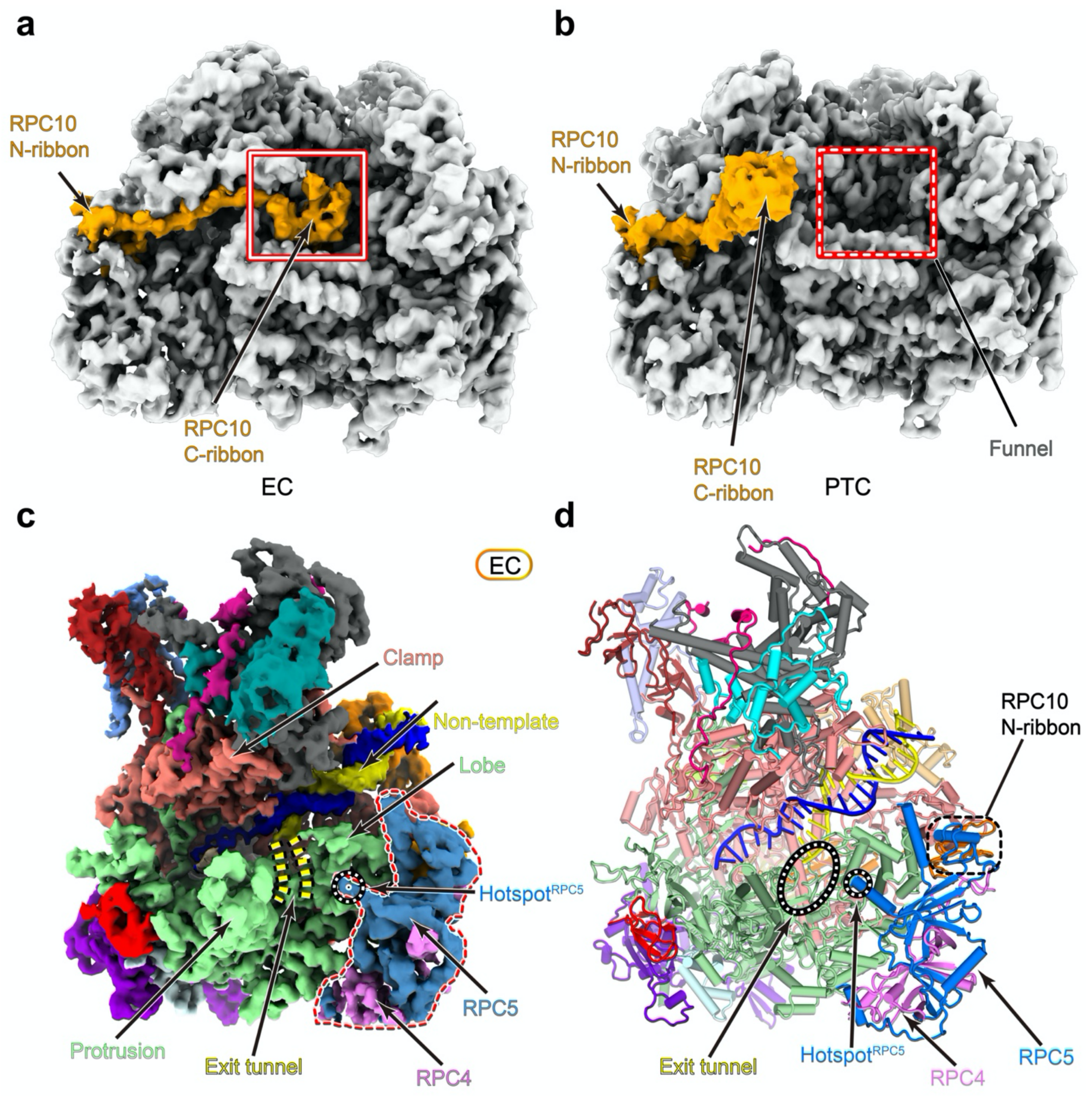

Supplementary Figure 4. Cryo-EM structure of the human Pol III EC.

(a) Cryo-EM map of human Pol III EC shows the C-ribbon of RPC10 in the funnel. (b) Cryo-EM map of human Pol III PTC shows that the C-ribbon of RPC10 is flipped out of the funnel. The funnel is indicated with dashed box. The N-ribbon of RPC10 is similarly positioned in EC and PTC. (c) Cryo-EM map of the human Pol III EC in this study. Critical regions are highlighted

and/or encircled with dashed lines. (d) Structural model of Pol III EC. The dashed circle indicates the putative non-template strand exit tunnel.

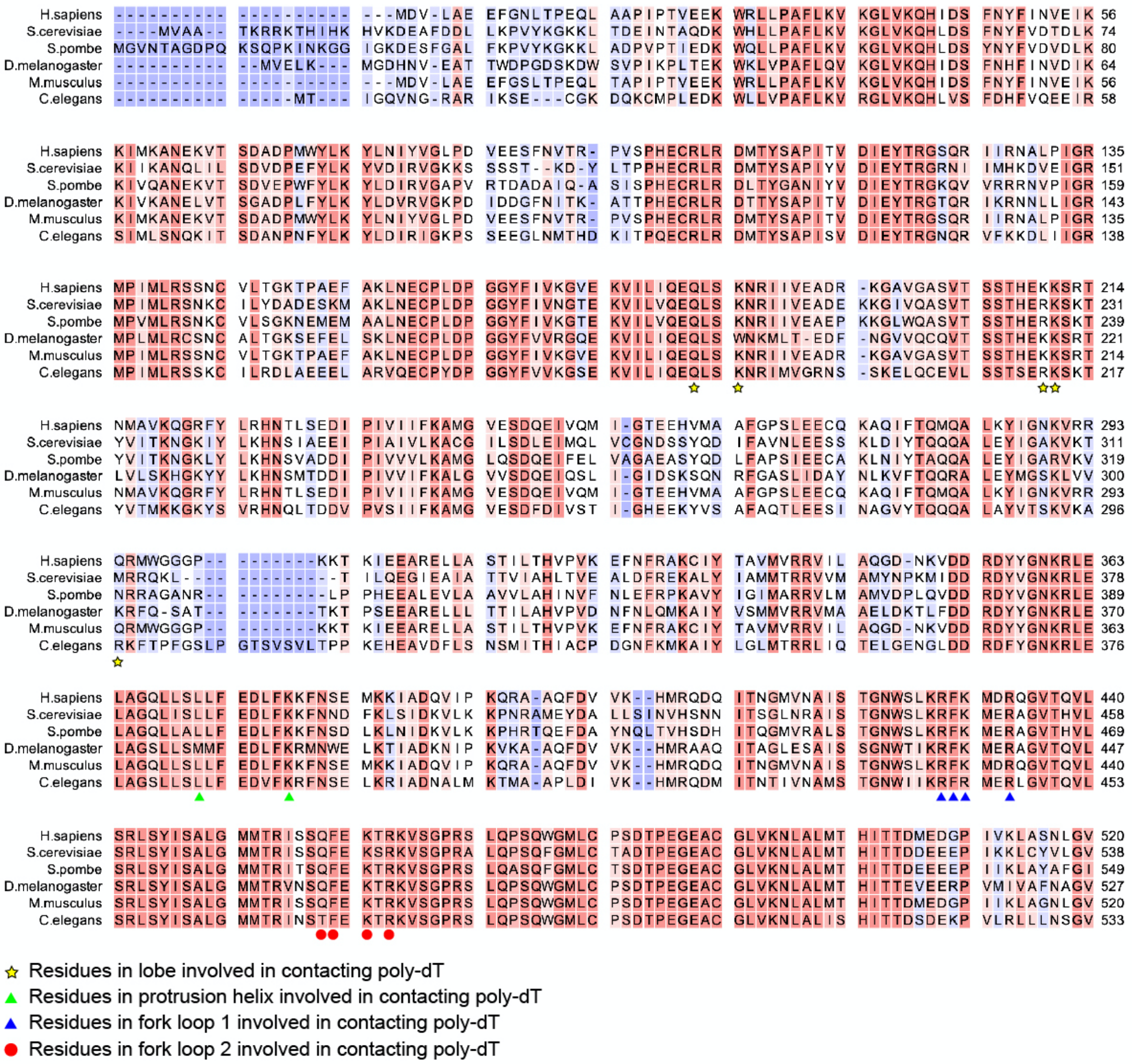

Supplementary Figure 5. Multiple sequence alignments of the N-terminus (1-520 in human Pol III) of RPC2 from model organisms.

Residues involved in poly-dT recognition are highlighted by different indicators.

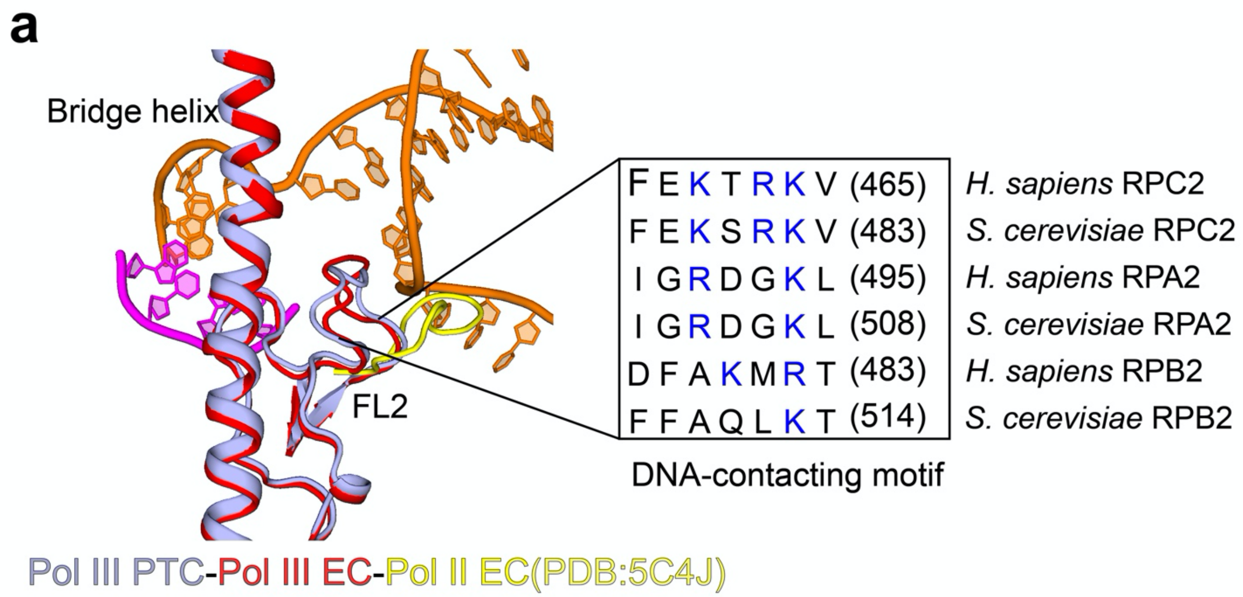

Supplementary Figure 6. Structural comparison of the exit tunnel of Pol I, Pol II, and Pol III.

(a) Structural differences of the human Pol III EC (red), PTC (grey), Pol II EC (PDB: 5C4J, yellow) around the transcription fork. The FL2 in both Pol III EC and PTC are in "open" states. Structure-based sequence alignment of the DNA-contacting motifs in the FL2 of Pol I/II/III from *Homo*

sapiens and *Saccharomyces cerevisiae* placed adjacent right panel. The positively charges residues are colored in blue. (b-c) Close-up views of the comparison of human Pol III PTC (grey) in this study with yeast Pol I EC (PDB:6HKO, blue) (b) and the yeast Pol II EC (PDB: 5C4J, yellow) (c). DNA and the exit tunnel are shown and the rest parts are omitted for clarity. Structural differences are indicated with arrows.

Supplementary Table 1. DNA sequences for RNA extension assay.

Name	Sequence (5' to 3')	Terminator	Figure
5S_T	GCCAAAGAAAAAGCCTACAGCACCCGGTATTCCCAGGCGGTCTCCCATCCACCAGTCAGCAATA	T5CT3	Fig. 5a
5S_NT	CACATTGGATGGGAGACCGCCTGGGAATACCGGGTGCTGTAGGCTTTTTCTTTGGC	T5CT3	Fig. 5a
5S^{DT}_T	GCCTACAGCACCCGGTATTCCCAGGCGGTCTCCCATCCACCAGTCAGCAATA	None	Fig. 5a
5S^{DT}_NT	CACATTGGATGGGAGACCGCCTGGGAATACCGGGTGCTGTAGGC	None	Fig. 5a
TS1^{T1}_T	GAAACAGCGGCTCTAGTCTGATCGTAAGCACCCGGTATTCCCAGGCGGCCAGTCAGCAATA	T1	Supplementary Fig. 1b
TS1^{T1}_NT	CACATCCGCCTGGGAATACCGGGTGCTTACGATCAGACTAGAGCCGCTGTTTC	T1	Supplementary Fig. 1b
TS1^{T3}_T	GAAACAGCGGCTCTAGTCTGAAATCGTAAGCACCCGGTATTCCCAGGCGGCCAGTCAGCAATA	T3	Supplementary Fig. 1b
TS1^{T3}_NT	CACATCCGCCTGGGAATACCGGGTGCTTACGATTTCAGACTAGAGCCGCTGTTTC	T3	Supplementary Fig. 1b
TS1^{T5}_T	GAAACAGCGGCTCTAGTCTGAAAAATCGTAAGCACCCGGTATTCCCAGGCGGCCAGTCAGCAATA	T5	Supplementary Fig. 1b
TS1^{T5}_NT	CACATCCGCCTGGGAATACCGGGTGCTTACGATTTTTCAGACTAGAGCCGCTGTTTC	T5	Supplementary Fig. 1b
TS1^{T7}_T	GAAACAGCGGCTCTAGTCTGAAAAAAATCGTAAGCACCCGGTATTCCCAGGCGGCCAGTCAGCAATA	T7	Supplementary Fig. 1b
TS1^{T7}_NT	CACATCCGCCTGGGAATACCGGGTGCTTACGATTTTTTTCAGACTAGAGCCGCTGTTTC	T7	Supplementary Fig. 1b
TS1$^{\Delta T}$_T	TCGTAAGCACCCGGTATTCCCAGGCGGCCAGTCAGCAATA	None	Supplementary Fig. 1b
TS1$^{\Delta T}$_NT	CACATCCGCCTGGGAATACCGGGTGCTTACGA	None	Supplementary Fig. 1b
TS1$^{\Delta T+5}$_T	(GTCTG)TCGTAAGCACCCGGTATTCCCAGGCGGCCAGTCAGCAATA	None	Supplementary Fig. 1b
TS1$^{\Delta T+5}$_NT	CACATCCGCCTGGGAATACCGGGTGCTTACGA(CAGAC)	None	Supplementary Fig. 1b

5S_T and 5S_NT represent the template and non-template strands of 5S DNA templates. The poly-dTs are underlined. TS1$^{\Delta T}$ represents the deletion of poly-dT in TS1 and TS1$^{\Delta T+5}$ represents the addition of five non-dT nucleotides to the non-templated strand of the TS1$^{\Delta T}$.
