# Supplementary material for: Structural insights into RNA polymerase III-mediated transcription termination through trapping poly-deoxythymidine
Source: Nat Commun. 2021 Oct 21;12:6135. doi: 10.1038/s41467-021-26402-9 (PMC8531034; doi:10.1038/s41467-021-26402-9)
Supplement: Supplementary file 2 — Description of Additional Supplementary Files [file 41467_2021_26402_MOESM2_ESM.pdf]

Description of Additional Supplementary Files

Supplementary Movie 1: The cryo-EM map and structural model of the Pol III pre-termination complex. Colour scheme is similar to that in Fig. 1b.

Supplementary Movie 2: The cryo-EM map and structural model of the Pol III elongation complex. Colour scheme is similar to that in Extended data Fig. 4a.

Supplementary Movie 3: A model of transition from elongation complex to pre-termination complex. Colour scheme is similar to that in Fig. 1b. Key residues in FL1 and FL2 are shown as red and violet sticks, respectively.
